# Detection of Sub-Clinical CWD Infection in Conventional Test-Negative Deer Long after Oral Exposure to Urine and Feces from CWD+ Deer

**DOI:** 10.1371/journal.pone.0007990

**Published:** 2009-11-24

**Authors:** Nicholas J. Haley, Candace K. Mathiason, Mark D. Zabel, Glenn C. Telling, Edward A. Hoover

**Affiliations:** 1 Department of Microbiology, Immunology, and Pathology, College of Veterinary Medicine and Biomedical Sciences, Colorado State University, Fort Collins, Colorado, United States of America; 2 Department of Molecular Biology and Genetics, University of Kentucky, Lexington, Kentucky, United States of America; Ohio State University, United States of America

## Abstract

**Background:**

Chronic wasting disease (CWD) of cervids is a prion disease distinguished by high levels of transmissibility, wherein bodily fluids and excretions are thought to play an important role. Using cervid bioassay and established CWD detection methods, we have previously identified infectious prions in saliva and blood but not urine or feces of CWD+ donors. More recently, we identified very low concentrations of CWD prions in urine of deer by cervid PrP transgenic (Tg[CerPrP]) mouse bioassay and serial protein misfolding cyclic amplification (sPMCA). This finding led us to examine further our initial cervid bioassay experiments using sPMCA.

**Objectives:**

We sought to investigate whether conventional test-negative deer, previously exposed orally to urine and feces from CWD+ sources, may be harboring low level CWD infection not evident in the 19 month observation period. We further attempted to determine the peripheral PrP^CWD^ distribution in these animals.

**Methods:**

Various neural and lymphoid tissues from conventional test-negative deer were reanalyzed for CWD prions by sPMCA and cervid transgenic mouse bioassay in parallel with appropriate tissue-matched positive and negative controls.

**Results:**

PrP^CWD^ was detected in the tissues of orally exposed deer by both sPMCA and Tg[CerPrP] mouse bioassay; each assay revealed very low levels of CWD prions previously undetectable by western blot, ELISA, or IHC. Serial PMCA analysis of individual tissues identified that obex alone was positive in 4 of 5 urine/feces exposed deer. PrP^CWD^ was amplified from both lymphoid and neural tissues of positive control deer but not from identical tissues of negative control deer.

**Discussion:**

Detection of subclinical infection in deer orally exposed to urine and feces (1) suggests that a prolonged subclinical state can exist, necessitating observation periods in excess of two years to detect CWD infection, and (2) illustrates the sensitive and specific application of sPMCA in the diagnosis of low-level prion infection. Based on these results, it is possible that low doses of prions, e.g. following oral exposure to urine and saliva of CWD-infected deer, bypass significant amplification in the LRS, perhaps utilizing a neural conduit between the alimentary tract and CNS, as has been demonstrated in some other prion diseases.

## Introduction

Chronic wasting disease (CWD) is an efficiently transmitted prion disease of cervids (e.g. deer, elk, and moose), and is the only known prion disease affecting free-ranging, non-domestic animals. The origins of CWD are uncertain, but the disease has been present in wild cervid populations of northern Colorado and southern Wyoming for at least 40 years [1], [2]. Since its discovery, CWD has been identified in captive and free-ranging cervids in 15 states, 2 Canadian provinces, and Korea [3]. As surveillance efforts have intensified, CWD has been detected in areas previously thought to be free of infection, including recent discoveries in West Virginia, New York, and Michigan [4], [5], [6]. The prevalence of CWD varies across North America, but can be as high as 30% in some areas of Colorado or in captive populations [7], [8].

Although the mechanisms of CWD transmission are incompletely understood, there is evidence inferring that infection is transmitted horizontally via saliva and urine [9], [10], and may be acquired from the environment [11], [12], [13]. With the expanded recognition of the disease across the continental United States, it is also likely that substantial human exposure has occurred. However, because of an apparently strong species barrier [14], [15] and the as yet incompletely understood natural routes and kinetics of CWD transmission, the magnitude and consequence of this exposure remain speculative.

While infectious CWD prions have been detected in saliva, blood, urine, and feces (i.e. prionsialia, prionemia, prionuria, and prionochezia) conventional CWD diagnostic assays (e.g. western blotting, immunohistochemistry, and enzyme-linked immunosorbent assay) have been unable to identify PrP^CWD^ in these materials [9], [10], [16], [17]. In the same vein, the early and antemortem identification of subclinically infected individuals requires demonstration of PrP^CWD^ in biopsied lymphoreticular system (LRS) tissues [18], [19], [20], [21]. Improving the sensitivity of low level of CWD prion detection in excreta or at early sites of accumulation, may allow for earlier antemortem diagnosis and a stronger estimate of prevalence [17], [22], [23], [24], [25], an approach which could also aid in detection of human transmissible spongiform encephalopathies (TSE's).

Apart from inoculation of susceptible hosts (bioassay), only *in vitro* amplification by serial protein misfolding amplification (sPMCA) [17], [26], [27], [28] offers the potential for comparable sensitivity. Here we have employed both of the latter methods to demonstrate low levels of infectious prions in clinically normal, conventional assay-negative white-tailed deer orally exposed 19 months previously to urine and feces from CWD+ deer.

## Materials and Methods

### Ethics Statement

All animals were handled in strict accordance with good animal practice as defined by relevant national and/or local animal welfare bodies, and all animal work was approved by Colorado State University Animal and Care Use Committee (ACUC approval number 08-175A-01).

### Infected Cervids

Five white-tailed deer (*Odocoileus virginianus*) were orally inoculated with urine (50ml total volume) and feces (50g total volume) from CWD+ donor deer. The deer were monitored for 19 months post inoculation (pi), during which time they remained asymptomatic. At 19 months pi, due to limitations in holding space imposed by other ongoing studies, the animals were euthanized, necropsied, and brain and lymphoid tissues examined for PrP^CWD^ by western blotting (WB) and immunohistochemistry (IHC) [9]. The sources of the urine and feces used for inoculation were three terminally-ill mule deer (*Odocoileus hemionus*) of unknown PrP genotype. Three of the inoculated deer were homozygous for glycine (i.e. G/G) at cervid PrP position 96 (Deer #'s 134, 141, and 150), while two were heterozygous at that location (Deer #'s 111 and 124), with alleles encoding for both glycine and serine (G/S). Tissues from all five animals were negative for PrP^CWD^ by WB and IHC and the animals were thus considered CWD-negative. The same tissue sets were collected from positive and negative control animals, including a deer inoculated intracranially (IC) with CWD+ brain (deer #106, G/S at position 96), two deer inoculated *per os* (PO) with saliva from CWD+ deer (deer #113 and 122, both G/G at position 96) and three deer inoculated by the IC and PO routes with brain homogenate from a CWD-naive deer (deer # 103 and 123, both G/G at position 96 and deer #4488 – G/S at this loci). Each deer was necropsied using fresh necropsy instruments, and all tissue samples were frozen at −70°C until use. All animals were maintained in accord with Colorado State University IACUC guidelines.

### Study Samples and Preparation

Obex, vagal nerve, intermediolateral spinal cord segments, sections of ileum, and a number of lymphoid tissues, including tonsil and retropharyngeal, mesenteric, mediastinal, and ileocecocolic lymph nodes, were collected at necropsy and frozen at −70°C. In initial sPMCA and bioassay experiments, obex and retropharyngeal lymph node (RLN) tissues from individual animals were pooled, as both these tissues have proven sensitive in the identification of PrP^CWD^ at various stages of infection [22], [29], [30], [31], [32], [33]. In later serial PMCA (sPMCA) experiments, specific neural and LRS tissues were analyzed individually. In each case, a fifty milligram section of each tissue was washed twice in phosphate-buffered saline (PBS), then homogenized and prepared as a 1% solution (w/v) in PBS using a FastPrep™ tissue homogenizer for 40s at power setting 6.5. All tissues were prepared in individual microcentrifuge tubes and homogenized in parallel in the same machine concurrent with controls.

### Cervid PrP Transgenic Mice

Tg[CerPrP] line 5037 (*tg5037*) mice were generated in the Telling laboratory at the University of Kentucky [34]. These mice express, both centrally and peripherally, an allelic variant of the prion gene possessed by Rocky Mountain Elk (*Cervus elaphus*), coding for glutamic acid at position 226 of the cervid prion protein. All mice were screened at weaning for the presence of the cervid *PRNP* transgene by conventional and real-time PCR. Mice testing negative for PrP^CWD^ at the completion of bioassay studies were rescreened to confirm the presence of cervid transgene. Mice were inoculated and maintained in accord with Colorado State University IACUC guidelines.

### Serial Protein Misfolding Cyclic Amplification (sPMCA)

Tissue homogenates from CWD-exposed deer, negative by WB, IHC, and ELISA, were assayed for PrP^CWD^ by sPMCA. In initial experiments, CWD+ obex/RLN homogenate from a white-tailed deer IC-inoculated with CWD+ brain (deer #106) was used as a positive control, while tissue preparations from two sham-inoculated deer (#103 and 123) and untreated *tg5037* mice were used as negative controls. In later experiments, a tissue set from a deer orally inoculated with CWD+ brain (deer #148) was selected for use as positive controls; negative control animals remained unchanged. All test samples were prepared in parallel with tissue-matched positive and negative controls as a 1% homogenate in PBS as described above and subsequently spiked into normal brain homogenate for amplification as described previously [17], [28], [35]. Normal brain homogenate (NBH), the substrate for prion conversion *in vitro*, was prepared from *tg5037* mice in a room that had not previously been used for prion research. Following euthanasia and perfusion with 5mM EDTA in phosphate-buffered saline (PBS), whole brain was collected from naïve *tg5037* mice and placed on ice. Brain homogenates were prepared as a 10% (w/v) solution in PMCA buffer (1% triton-X 100 [v/v], 5mM EDTA, and 150mM NaCl in PBS adjusted to a pH of 7.2) with the addition of Complete Protease Inhibitors (Roche Pharmaceuticals, Indianapolis, IN) using a dounce homogenizer. Homogenates were then centrifuged for 1 minute at 2000rpm and the supernatant frozen in single-experiment aliquots at −70°C in a “prion-free” room until use in PMCA. Fifteen µl of test or control tissue homogenate was added to 45µl of NBH and assayed, in parallel and in adjacent wells of a 96-well plate (USA Scientific, Ocala, FL); along with normal brain homogenate prepared from unexposed *tg5037* mice as additional, unseeded negative controls. Plates were then sonicated using an ultrasonic processor (Misonix, Farmingdale, NY) and incubated at 37°C. Sonication parameters were set at 40s bursts at power level 7.0, followed by 30 minutes of incubation. Ninety six cycles of sonication were performed over 48 hours, with a 10µl aliquot transferred to 50µl of fresh NBH for serial amplification. Following three rounds of amplification, samples were evaluated by western blotting, as described below, for the presence of PrP^CWD^. Brain homogenates from all mice testing negative for PrP^CWD^ in bioassay experiments were likewise analyzed to increase detection sensitivity. In analysis of individual cervid tissue samples, occasional differences were noted in the round in which amplification was initially observed. For this reason, we tallied the number of successive positive rounds for each sample in each repetition for semi-quantitative analysis. Over the course of sPMCA experiments, multiple NBH preparations were used, and each test or control sample was evaluated at least three times for repeated verification of results.

### Mouse Bioassays

Four groups of *tg5037* mice (n = 8/group) were anesthetized with ketamine and xylazine and inoculated intracerebrally into the left parietal lobe with 30µl of 1% obex/RLN homogenate. Positive control mice were inoculated with obex and RLN from a deer IC-inoculated with CWD+ brain (deer #106), while a single negative control group was inoculated with combined obex and RLN homogenates from two sham-inoculated deer (deer #103 and 123). Obex and RLN homogenates from two deer orally inoculated with urine and feces (deer #134 and 150) were selected for bioassay experiments based on their apparent amplification ability in sPMCA experiments (below). Incubation time was defined as the number of days from inoculation to the onset of clinical signs of transmissible spongiform encephalopathy (TSE), as previously described [36]. Animals were euthanized when either signs of clinical TSE or distress were evident. Brain harvested at necropsy was divided longitudinally, with one hemi-section prepared for evaluation by western blotting and sPMCA and the remaining hemi-section fixed in 10% neutral-buffered formalin for immunohistochemical analysis

### Western Blotting (WB)

Brain hemi-sections for WB and sPMCA were initially prepared as a 10% (w/v) solution in PMCA buffer. Eleven µl of sample homogenate were mixed with 7µl of sample buffer (0.1% [v/v] triton-X 100 and 4%(w/v) SDS in PBS) and digested with 2µl proteinase-K at 500µg/ml (final concentration: 50µg/ml) for 20′ at 37°C followed by 10′ at 45°C. Seven µl of 4× running buffer were then added to the sample, followed by denaturation for 5′ at 95°C. Twenty µl of this preparation were run on a pre-cast 12% SDS-PAGE gel (Invitrogen) in a Bio-Rad electrophoresis apparatus for 2 hours at 120mV. Samples were then transferred to a PVDF membrane (Millipore) for 1 hour at 110mV in a Bio-Rad transfer apparatus. PVDF membranes were subsequently blocked for 1 hour in 5%(w/v) powdered milk in 0.2% Tween-20 in tris-buffered saline (TBST), followed by application of the primary antibody, BAR224-HRP, diluted 1∶20,000 in TBST with 5% powdered milk, for 1 hour. Following washing, immunoreactivity was detected using an enhanced chemiluminescent detection system (ECL-plus, Amersham Biosciences) in a LAS 3000 imaging system. (Fuji Photo Film, Fuji Inc, Valhalla, NY)

### Neuropathology and Immunohistochemistry (IHC)

Brain hemi-sections were fixed in formalin overnight, treated with 88% formic acid for one hour, washed in tap water and then stored in 60% ethanol prior to paraffinization. Paraffin-embedded tissue sections (6µm) were mounted onto positively charged glass slides, deparaffinized, and rehydrated through graded ethanol. To enhance detection, tissues were subjected to Heat Induced Epitope Retrieval (HIER) using an automated antigen-retrieval system (Retriever™) and a proprietary buffer solution (DakoCytomation Target Retrieval Solution, DAKO, Hamburg, Germany). Tissues were then stained with an automated immunostainer, using polyclonal PrP antibody R-505 as the primary antibody (a gift from Dr. Jan Langeveld, Central Veterinary Institute of Wageningen University), The Netherlands) at a 1∶500 final dilution, followed by secondary application of a universal anti-rabbit polyclonal antibody conjugated to horseradish peroxidase (HRP). Detection was completed using HRP-mediated hydrogen peroxide immunostaining (AEC+, DAKO), with hematoxylin as a counterstain.

## Results

To evaluate CWD-exposed, yet IHC-, and WB-negative, deer for subclinical CWD infection, we pooled obex and retropharyngeal lymph node (RLN) tissues in an effort to enhance detection sensitivity. Pooled tissues from individual animals were initially analyzed via serial PMCA (sPMCA) with results subsequently confirmed in two of these individuals using bioassay in cervidized *tg5037* mice. Serial PMCA was then used to analyze individual nervous and LRS tissues to estimate the mechanism of CNS invasion in animals orally exposed to presumed low doses of CWD prions that may be present in urine and feces.

### Serial PMCA Amplification of PrP^CWD^ from Tissues of Deer Orally Exposed to Urine and Feces

**Figure 1:**
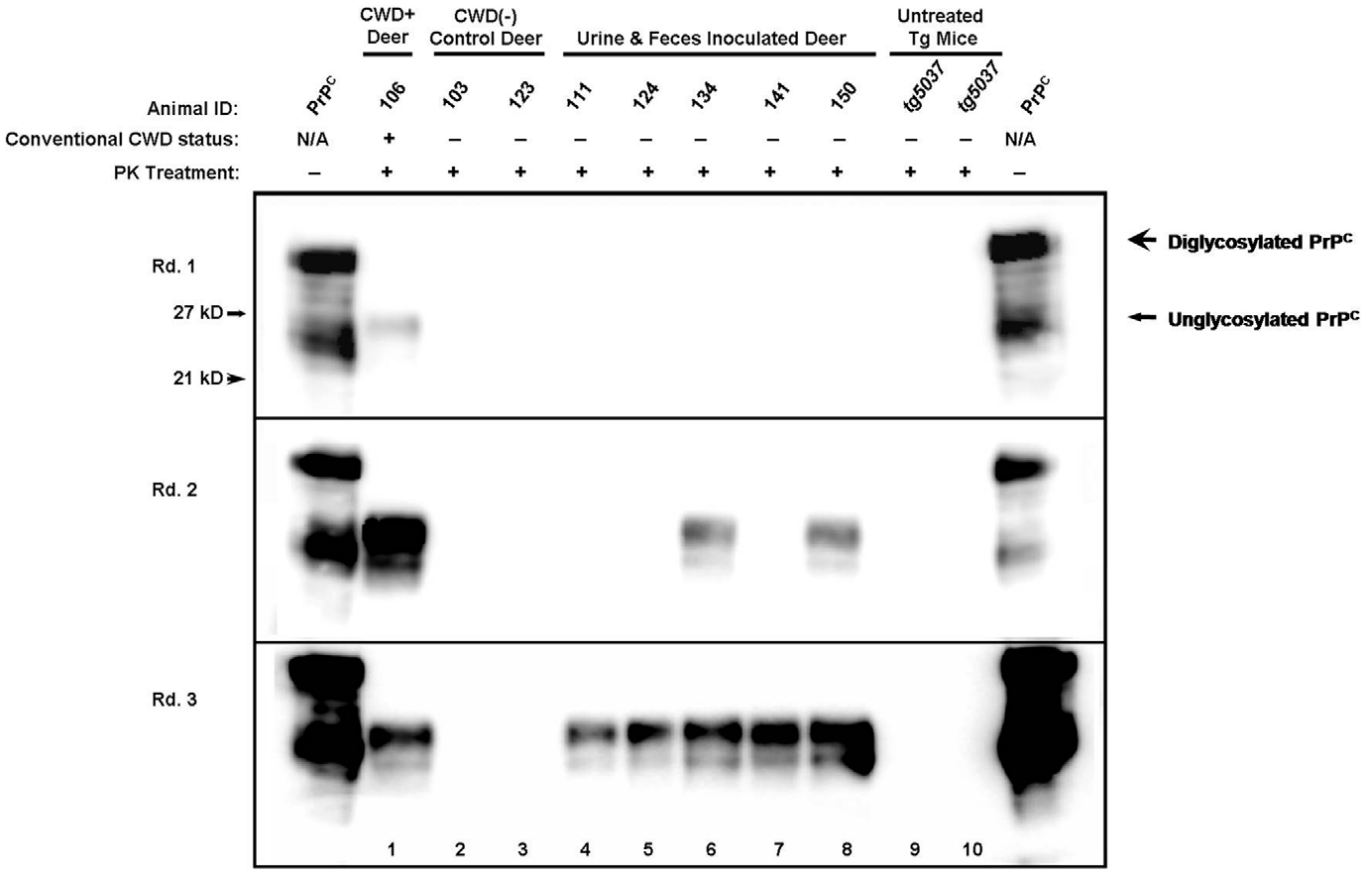
Serial PMCA amplification of CWD prions in WB and IHC negative deer. Conventionally negative tissues from deer orally exposed to urine and feces from CWD+ sources (Deer #'s 111, 124, 134, 141, and 150, lanes 4–8) amplified PrP^CWD^ after 2–3 rounds of PMCA, as did positive control tissues from deer #106 (lane 1). Tissue samples from two sham-inoculated deer (#103 and 123, lanes 2 and 3) and two untreated *tg5037* mice (lanes 9 and 10) failed to amplify PrP^CWD^ in three rounds of sPMCA.

Obex and RLN tissues collected at necropsy from experimentally exposed deer were prepared as a 1% solution in PBS and subjected to three rounds of sPMCA as described. In our experience, three rounds of amplification permits a 2000 to 4000-fold increase in sensitivity as compared to traditional western blotting detection, while maintaining 100% specificity [17], [28]. In three independent experiments, obex and RLN homogenates from individual deer that had been orally inoculated with urine and feces from a CWD+ source (deer #'s 111, 124, 134, 141 and 150) consistently amplified PrP^CWD^ by sPMCA. Positive control tissues from an IC-inoculated deer (deer #106) also successfully amplified PrP^CWD^ through each round, while concurrently run control tissue homogenates from CWD-negative brain-inoculated deer (deer #'s 103 and 123) or naïve *tg5037* mice were negative by sPMCA. (Figure 1)

Based on these findings, tissues from two white-tailed deer with the greatest apparent *in vitro* amplification ability (deer #134 and #150, Fig. 1), along with tissue-matched controls, were selected for further evaluation by *tg5037* mouse bioassay. Groups of mice were inoculated with obex/RLN homogenates, monitored for clinical signs of prion infection, and euthanized when terminal disease was apparent. Brains from inoculated mice were evaluated for PrP^CWD^ by WB, IHC and, when negative by these assays, also by sPMCA.

### Authentic Prion Infectivity Identified in tg5037 Mouse Bioassay

**Figure 2:**
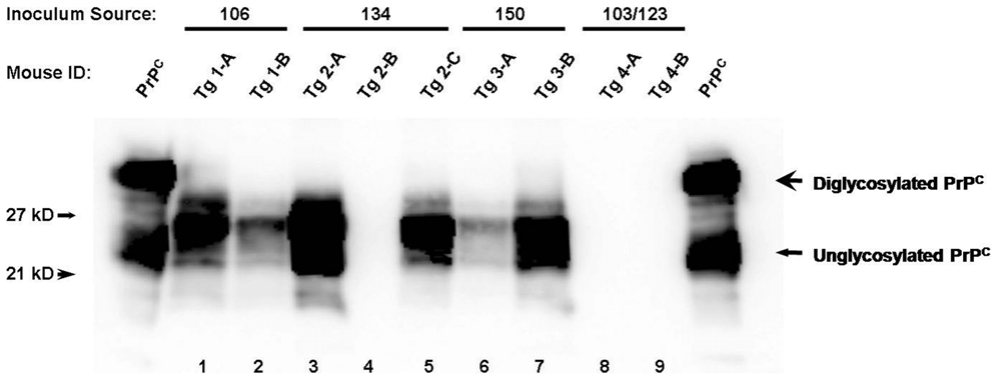
*Tg5037* mouse bioassay results. Kaplan-Meyer curve demonstrating prolonged incubation periods in mice inoculated with tissues from deer #134 and 150 as compared to mice inoculated with tissues from deer testing positive by conventional assays.

**Figure 3:**
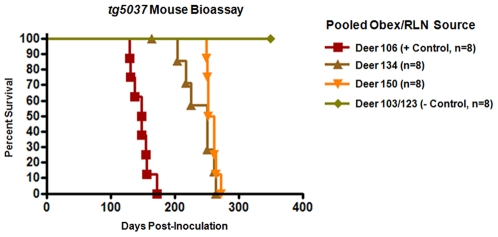
Western blot detection of PrP^CWD^ in mouse CNS tissues. Except for a single mouse (mouse Tg 2-B, lane 4), all mice inoculated with tissues from deer #134 and 150 succumbed to prion disease (lanes 3–7), as did mice inoculated with CWD+ deer #106 (lanes 1 and 2). Mice inoculated with tissues from sham-inoculated deer showed no evidence of PrP^CWD^ by western blot (lanes 8 and 9).

In a group of 8 mice inoculated with obex/RLN homogenates from deer #134, 7 of the 8 mice developed clinical signs consistent with TSE, including progressive ataxia and weight loss, by 264 days post inoculation (dpi). WB and IHC confirmed the presence of PrP^CWD^ in each of these seven mice. The remaining mouse in this group died of intercurrent disease at 161 dpi. This mouse showed no evidence of clinical TSE and was negative for PrP^CWD^ by both IHC and WB. (Fig. 2, mouse #2-B) In a second group of mice, inoculated with tissues from deer #150, 8 of 8 mice developed similar clinical signs of TSE infection and were confirmed PrP^CWD^-positive by WB and IHC by 272 dpi. All mice inoculated with brain and RLN homogenates from positive control deer #106 developed clinical TSE and were PrP^CWD^-positive by 173 dpi, whereas negative control mice remained healthy until euthanasia at 340+ dpi, at which time they were also negative for PrP^CWD^ by WB and IHC. (Fig. 3 and Table 1)

**Table 1:** Summary of immunohistochemistry (IHC), western blot (WB), and serial PMCA results and *Tg5037* mouse bioassay of combined obex/RLN homogenates.

Mouse Bioassay				
Inoculum	IHC +'s	WB +'s	sPMCA +'s	Incubation Period
106	8/8	8/8	N/A	148 (11)
134	7/8	7/8	1/1	238 (23)
150	8/8	8/8	N/A	259 (8)
103/123	0/8	0/8	0/8	340+

Mean incubation periods with standard deviations in parentheses; numerators indicate number of animals testing positive over total number tested. For PMCA, only those animals testing negative by IHC and WB were assayed. N/A: not assayed.

### Biochemical Confirmation of Bioassay

**Figure 4:**
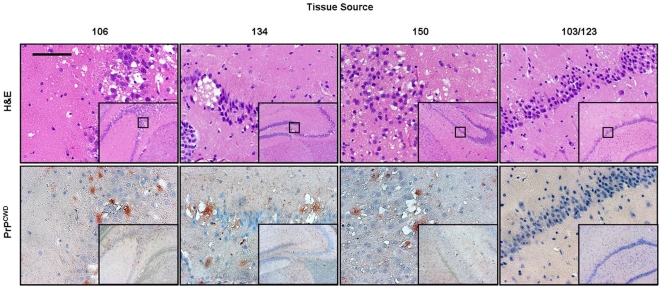
Spongiform degeneration and PrP^CWD^ in the hippocampus of inoculated mice. Vacuolated neurons and spongiform degeneration of the neuropil characteristic of TSE demonstrated by H&E staining and co-localization of PrP^CWD^ florid plaques in the hippocampus of mice inoculated with tissues from urine and feces exposed and positive control deer. Brains of mice inoculated with tissues from sham-inoculated deer showed no evidence of spongiform degeneration or PrP^CWD^ immunostaining. Anti-prion polyclonal antibody R-505 was used as the primary antibody. (Measure bar, 50 µm)

Western blot glycoform patterns were typical of CWD in Tg[CerPrP] *tg5037* mice, spanning 21–27 kD following proteinase-K digestion and dominated by a di-glycosylated PrP^CWD^ isoform. Representative WB's from each group are shown in Figure 2. Immunohistochemistry demonstrated a relatively narrow distribution of PrP^CWD^ within the hippocampus of affected mice, colocalizing with vacuolization and spongiform degeneration of the neuropil. There was no apparent relationship between deposition pattern, lesion intensity, and source inoculum. (Fig. 4)

### Serial PMCA Identification of PrP^CWD^ in an Asymptomatic Mouse

**Figure 5:**
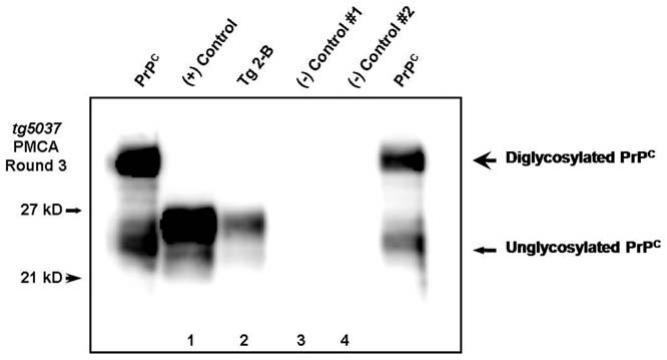
Serial PMCA detection of CWD prions in inoculated mice. Brain from a single *tg5037* mouse (mouse Tg 2-B) was WB and IHC negative yet amplified PrP^CWD^ after three rounds of sPMCA (lane 2), as did a positive control mouse (lane 1). Mice inoculated with tissues from sham-inoculated deer failed to amplify PrP^CWD^ (lanes 3 and 4).

Negative control mice and a single test mouse expiring with intercurrent disease at 161dpi, all of which were found negative for PrP^CWD^ by WB and IHC, were further evaluated using sPMCA over three rounds of amplification. In three repeated sPMCA experiments, a subclinical prion infection was confirmed in the remaining mouse inoculated with tissue from deer #134 (mouse #Tg 2-B). None of the brains from mice inoculated with negative control tissues amplified PrP^CWD^, while positive control mice amplified PrP^CWD^ successfully over three rounds. (Fig. 5)

### Analysis of Individual Neural and Lymphoid Tissues by sPMCA

To further examine the terminal distribution of CWD prions following oral exposure to presumed low concentrations of infectious prions in excreta, we analyzed individual tissue samples from control and test deer using sPMCA. Positive control tissues in these experiments were from two deer inoculated PO with saliva from CWD+ deer (deer #113 and 122), while identical tissue sets were collected from negative control deer #103, 123, and 4488. By analyzing obex and retropharyngeal tissues individually, PrP^CWD^ amplification was found to occur exclusively in obex preparations from study animals. The levels of amplification observed were compared to serial dilutions of positive control tissue, and correlated to an approximately 20–40 fold dilution of 1% homogenate of deer #106 obex in NBH (data not shown). These results were surprising given that it has been reported that PrP^CWD^ is commonly found to accumulate in the retropharyngeal lymph node, and other lymph nodes of the alimentary tract, prior to its appearance in the obex in experimental oral [31] and natural CWD infections [8], [30]. We therefore sought to determine whether other lymphoid tissues were correspondingly bypassed in these deer.

**Figure 6:**
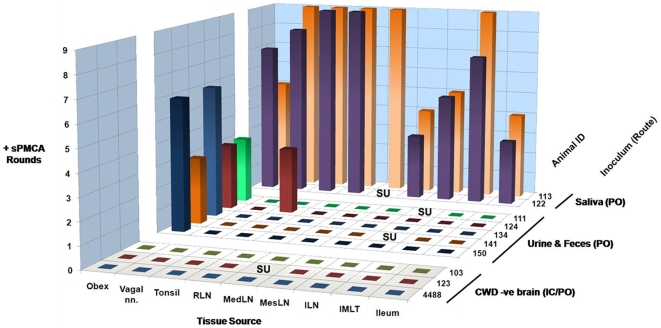
Serial PMCA detection or PrP^CWD^ of neural and lymphoid tissues of deer exposed orally to urine and feces of CWD-positive deer. For each tissue, the number of sPMCA rounds producing a positive result was tabulated for three independent experiments of three rounds each. Samples appearing as positive in round 2 continued to be positive in round 3, and thus over the course of three experiments, were positive a total of 6 out of 9 rounds (e.g. obex samples from deer 134 and 150). Samples becoming positive in the first round were succeeded by positivity in rounds 2 and 3, and thus were positive in 9 out of 9 rounds (e.g. tonsil and RLN samples from deer 122 and 113). As would be expected, the greatest number of positive results was observed in the positive control tissues, with some variance among tissues and between animals. Deer orally exposed to urine and feces demonstrated PrP^CWD^ amplification almost exclusively from the obex; the terminal tonsil collection from a single animal also was positive. By contrast, negative control tissues failed to amplify PrP^CWD^. RLN: retropharyngeal lymph node; MedLN: mediastinal lymph node; MesLN: mesenteric lymph node; ILN: ileocecocolic lymph node; IMLT: Intermediolateral spinal cord tract; SU: sample unavailable.

In an attempt to understand if a strictly neural route of CNS invasion might have occurred, we performed sPMCA on the mesenteric, mediastinal, and ileocecocolic lymph nodes, tonsils, vagus nerve, intermediolateral spinal cord segments, and ileum from the above 5 urine/feces exposed deer and from positive and negative control deer. Surprisingly, while PrP^CWD^ was amplified from a terminal tonsil biopsy of a single study deer, neither vagus nerve, nor the retropharyngeal, mesenteric, mediastinal or ileocecocolic lymph nodes were positive for PrP^CWD^ by sPMCA in any of the deer orally exposed to urine and feces from CWD+ donors (Figure 6). As might be expected, protease-resistant prion protein was amplified from both neural and lymphoid tissues from the positive control deer, while no amplification was observed in corresponding tissues from negative control deer or multiple unseeded NBH controls examined concurrently and on the same plate as positive samples.

## Discussion

The salient feature of chronic wasting disease is its facile transmission among captive and free-ranging cervids. We have previously demonstrated infectious prions in the saliva and blood using cervid bioassay [9], [10]. However, we were unable to identify PrP^CWD^ in the tissues of deer orally exposed to combined urine and feces, even though our later studies employing Tg[CerPrP] mouse bioassay demonstrated very low levels of prion infectivity (surmised by long incubation/survival periods) in the urine of some CWD+ deer [17]. In the current study, the enhanced sensitivities of sPMCA and intracerebral inoculation of cervid PrP-*tg5037* mice have led us to conclude that at 19 months post inoculation, low levels of amplifiable and infectious CWD prions were in fact present in the brains of the exposed, yet asymptomatic and conventional assay negative, deer from our original oral bioassay studies [9], [10], thereby inferring that low levels of prion infectivity were present in the urine/feces inocula. Whether the apparent low concentrations of prions amplifiable from the obex of the urine/feces recipients could represent a persistent non-pathogenic prion carrier state, or rather (perhaps more likely) indicates that an observation period far exceeding 19 months would be required to reveal ultimately pathogenic prion infections in these animals, remains undetermined.

Our initial bioassay deer were inoculated with both urine and feces, thus our present findings cannot identify the specific excreta involved in CWD transmission. It seems likely, however, that both excreta may play an important role in the horizontal transmission of CWD, given the later demonstration of prion infectivity in urine [17] and feces [16] of CWD+ deer using transgenic mouse bioassay. Further experiments, employing cervid bioassay of separated excreta and sPMCA are underway to better answer this question.

In the initial sPMCA and bioassay experiments, to maximize use of available *tg5037* mice, we analyzed homogenates of combined neural (obex) and lymphoreticular (LRS) tissues for the CWD prion protein. Information on the pathogenesis of CWD indicated that, following oral exposure, PrP^CWD^ is first detectable in lymphatic tissues draining the alimentary tract, especially retropharyngeal lymph node and Peyer's patches [8], [29], [30], [31], [37]. Thereafter, it is assumed that neural transport may be occur through anterograde ascension via myenteric or LRS sympathetic and parasympathetic neural networks to the central nervous system [37], paralleling the pathogenesis of sheep scrapie [38], [39]. Thus the earliest and most prominent CNS PrP^CWD^ accumulation is in the dorsal motor nucleus of the vagus in the obex [22], [30], [40], followed by centrifugal neural spread to peripheral sites. We were therefore surprised to find that PrP^CWD^ amplification was restricted to the obex of 4 out of 5 urine/feces inoculated deer. As with any assay, there is the potential for sample quality or the presence of inhibitors to affect test results, thereby explaining the absence of detectable PrP^CWD^ in the lymphoid tissues of urine and feces inoculated deer. However, the abundant successful amplification of PrP^CWD^ in lymphoid tissues of saliva-inoculated deer infers that a significant technical barrier to PrP^CWD^ amplification from lymph node is unlikely to explain the results. Thereby, we surmised that either CWD prions may have bypassed amplification in the LRS of these deer or were no longer present at detectable levels in the periphery. While these results are in contrast to the current concept of CWD pathogenesis [9], [30], [31], [37], absence of LRS involvement has been observed in natural cases of scrapie [41], [42], [43], [44] and bovine spongiform encephalopathy (BSE) of cattle and sheep [38], [39], [45], [46]. In scrapie-infected sheep, for example, the absence of scrapie amplification in the LRS as a result of host PrP genotype, limited dose exposure, tissue route/conduit, or prion strain selection are all supported by available evidence [41], [42], [43], [44], and may also be plausible in cases of CWD. The deer included in our study were of two PrP genotypes at position 96, expressing either G/G or G/S, both of which have been shown to be LRS competent in the pathogenesis of CWD, although slower pathogenesis has been linked to 96S [8], [19]. In naturally occurring cases of bovine spongiform encephalopathy, the BSE prion seems to exclusively utilize autonomic neural pathways for neuro-invasion, a defining characteristic of BSE [38], [39], [45], [46]. It is possible that CWD strains may eventually be uncovered with BSE-like phenotypes which could be selected for by low dose exposure. Nevertheless, there have been no reports of PrP^CWD^ detection solely in the CNS of infected deer. Thus the exact route by which CWD prions accessed the CNS of deer in the present study remains unidentified.

Detection of CWD prions in the present study samples relied heavily on sPMCA, with cervid transgenic mouse bioassay as a confirmatory assay. Given the risk of spurious positive results possible with multiple rounds of sPMCA, whether due to “spontaneous generation” or contamination [47], [48], [49], we took great care to minimize this risk using approaches that parallel those to reduce cross-contamination in nested PCR. These precautions took two forms: avoiding cross-contamination during sample collection and initial processing, and prevention of intra-experimental cross-contamination. Animals were necropsied in order of approximate level of infection (based on inoculation material and known tonsil biopsy results), beginning with negative controls, followed by animals exposed yet biopsy negative throughout the study, and finally exposed, biopsy positive animals. Fresh necropsy instruments were used for each necropsy, with the central nervous system tissues removed at the completion of the necropsy. All study and control samples were then prepared in parallel using identical equipment and reagents. Normal brain homogenate substrates were prepared and loaded in sPMCA plates in a laboratory not used for prion study using disposable or sterilized equipment. Negative controls included multiple samples from both sham-inoculated cervids and uninfected Tg[CerPrP]-*tg5037* mice. In addition, the number of amplification rounds was limited to three to help ensure specificity. Perhaps most importantly, the positive sPMCA results identified in brain and lymph node homogenates were confirmed by bioassay to provide support the conclusion that the sPMCA+ tissues did in fact harbor infectious prions.

In summary, we provide evidence for the presence of infectious prions in the brains of conventional prion-assay-negative deer orally exposed 19 months earlier to urine and feces from CWD-infected donor deer. This apparent low level of prion infection was amplified by sPMCA, confirmed by Tg[CerPrP] mouse bioassay, and detected only in the obex region of the brain. These results demonstrate the potential for CWD prion transmission via urine and/or feces, and highlight the application of more sensitive assays such as sPMCA in identification of CWD infection, pathogenesis, and prevalence.
